# Nationwide Survey in Greece about Knowledge, Risk Perceptions, and Preventive Behaviors for COVID-19 during the General Lockdown in April 2020

**DOI:** 10.3390/ijerph17238854

**Published:** 2020-11-28

**Authors:** Varvara A. Mouchtouri, Evagelia Agathagelidou, Kleovoulos Kofonikolas, Xanthi Rousou, Katerina Dadouli, Ourania Pinaka, Evi Agathocleous, Lemonia Anagnostopoulou, Chrysanthi Chatziligou, Eleni P. Christoforidou, Thekla Chalntoupi, Loukas Kalomoiris, Christina Kapoula, Vasiliki Kokkinou, Aggeliki Constantinides, Petros Konstantinou, Elina Kostara, Leonidas Kourentis, Anastasia Lantou, Georgios Lempidakis, Polixeni-Natalia Liasidi, Christos Michalakis, Dorothea Panagiotou, Freideriki Panteliadou, Vasileios Papadoulis, Grigorios Papantoniou, Maria Psatha, Dimitrios Ragias, Vera Ringa, Argyro Syrakouli, Angeliki Skoutari, Stella Stergiadou, Andreas Theodorou, Vasiliki Tzika, Areti Lagiou, Theodoros Dardavesis, Panagiotis Prezerakos, Christos Hadjichristodoulou

**Affiliations:** 1 Department of Hygiene and Epidemiology, Faculty of Medicine, University of Thessaly, 41222 Larissa, Greece; evagelia.ag.1967@gmail.com (E.A.); kleovmed@outlook.com (K.K.); xanrous@yahoo.com (X.R.); katerina1dad@gmail.com (K.D.); rpinaka@gmail.com (O.P.); eviagathocleous1@gmail.com (E.A.); lanagnost@uth.gr (L.A.); cchatziligou@gmail.com (C.C.); elchristof@uth.gr (E.P.C.); theklahalder@gmail.com (T.C.); loukasparker@gmail.com (L.K.); kapoulachris@gmail.com (C.K.); vasokokkinou@gmail.com (V.K.); aggelikiconst23@gmail.com (A.C.); petroskon18@gmail.com (P.K.); elkost@med.uth.gr (E.K.); leokourentis@med.uth.gr (L.K.); anastasia_lantou@hotmail.com (A.L.); lempidakis17@gmail.com (G.L.); natalialiasidi@gmail.com (P.-N.L.); xristosmix197@gmail.com (C.M.); info@dorothea.com.gr (D.P.); frider.pan@gmail.com (F.P.); papadoulisv@gmail.com (V.P.); papantoniougregory@gmail.com (G.P.); mariapsatham@gmail.com (M.P.); mjr9898@hotmail.gr (D.R.); vringa@bio.uth.gr (V.R.); argyrosyrakouli@gmail.com (A.S.); angiesk97@gmail.com (A.S.); stellastergiadou@gmail.com (S.S.); andtheodoroy@gmail.com (A.T.); vtzika@outlook.de (V.T.); xhatzi@med.uth.gr (C.H.); 2 Department of Public and Community Health, School of Public Health, University of West Attica, 12243 Athens, Greece; alagiou@uniwa.gr; 3 Department of Hygiene, Social-Preventative Medicine and Medical Statistics, School of Medicine, Aristotle University of Thessaloniki, 54124 Thessaloniki, Greece; dardaves@auth.gr; 4 Department of Nursing, University of Peloponnese, 22100 Tripoli, Greece; prezerpot@gmail.com

**Keywords:** coronavirus, COVID-19, knowledge, attitude, practices, behavior, Greece, risk perception, general population

## Abstract

Background: The aim of this study was to investigate the knowledge, attitudes, and practices of the Greek general population toward coronavirus disease 2019 (COVID-19) during the lockdown period in April 2020, to examine factors associated with misperceptions and to determine behavioral patterns that may require interventions. Methods: A cross-sectional study of the general Greek population (*N* = 1858) was conducted. A geographically stratified cluster sampling was implemented. A questionnaire was composed consisting of 35 questions. Data collection took place from 15 April to 2 May 2020. A random-digit dialing survey was conducted by 29 interviewers. Results: The majority of respondents (62.7%) answered ≥12/17 questions correctly. Participants aged 18–44 years, male gender, specific occupations (freelancer, unemployed, housewife, retiree) and those who sought information about COVID-19 from less than two sources received lower aggregated scores on knowledge questions. Regarding attitudes toward future vaccination, 18.9% declared that were against it, while 81.1% that they may consider or will be vaccinated. About 40% were not using a face mask and only 42% washed their hands appropriately. Conclusion: Adjusting information campaigns targeting especially people below 45 years of age can help to sensitize them and realise their role to control the spread. Further targeted surveys are needed to adjust/design prevention campaigns.

## 1. Introduction

In the current phase of the coronavirus disease 2019 (COVID-19) pandemic where a vaccine is not available, the success of non-pharmaceutical prevention strategies depends to a great extent on people’s behavior and their adherence to health advice. The European Centre for Disease Prevention and Control’s (ECDC) rapid risk assessment (24 September 2020) highlights that the observed increase in transmission levels in the European Union/European Economic Area countries and the United Kingdom indicate that “the *non-pharmaceutical interventions in place have not achieved the intended effect either because adherence to the measures is not optimal or because the measures are not sufficient to reduce or control exposure*” [1]. Population surveys can provide insights into people’s perception of risk, their practices, views on restrictions, misperceptions, information needs, and can further support the implementation of evidence-informed policies [2].

As of 15 November 2020, 220 countries areas or territories have reported to the World Health Organization (WHO) 53,507,282 confirmed cases and 1,305,164 confirmed deaths, while a total of 10,560,273 cases and 265,184 deaths have been reported in the European Union and European Economic Area and the United Kingdom [3,4]. On 23 March 2020 when a nationwide restriction of citizens’ movements was enforced, Greece had reported a total of 695 confirmed COVID-19 cases and 17 deaths [5]. The general lockdown was gradually lifted starting on 4 May, when a total of 2632 confirmed cases and 146 deaths had been reported. Before and during this lockdown period, prevention and control measures for COVID-19 were supported by health communication strategies through media campaigns, emergency alerts, live broadcasted daily briefings, a COVID-19 call line, and other methods [6]. Between May and July 2020, the number of new cases reported daily remained low (less than 20), while in August, when Greece resumed its tourism activities, the daily number rose to between 100 and 300 cases [7]. In September 2020, in addition to the information campaigns for the general public, prevention strategies targeted people returning from holidays, as well as educational institution activities. The ECDC’s rapid risk assessment report (24 September 2020) categorized Greece among the countries with stable trends; however, due to the strong increasing trend in intensive care unit admissions, the country may have the potential for a large resurgence [1]. The aim of this study was to investigate the Knowledge, Attitudes, and Practices (KAP) of the Greek population toward COVID-19 during the lockdown period in April 2020 to examine factors associated with misperceptions and to determine behavioral patterns that may require interventions.

## 2. Materials and Methods

A nationwide cross-sectional study was conducted. The representative target sample size that was needed in order to achieve the study objectives and sufficient statistical power was calculated with a sample size calculator RAOSOFT [8]. The sample size calculator arrived at 1537 participants, using a margin of error of ±2%, a confidence level of 95%, an 80% response rate, and 8,693,742 people (adult population of Greece). A geographically stratified sampling plan based on regional units, which are categorized as level 3 in accordance with the Nomenclature of Territorial Units for Statistics (NUTS) was applied to produce a representative sample. The sample size of each regional unit was calculated according to the population distribution of regional units in Greece. Moreover, the sample was also stratified based on gender and three proportionally equal to adult population age groups (“18–39” (34%), “40–59” (34%), “60+” (32%)). Data about population, age, and gender distributions were according to the 2011 census [9]. A questionnaire was composed considering the WHO Regional Office for Europe survey tool and guidance for rapid, simple, flexible behavioral insights on COVID-19 [2]. The questionnaire consisted of 35 questions about (1) demographic characteristics; (2) knowledge (COVID-19 transmission and symptoms); (3) perceptions (risks, effectiveness of preventive and control measures); (4) practices (prevention and control of infection); and (5) self-rating health and financial status. Most of the questions were closed, asking the respondent to evaluate by giving the answer in a quantitative value four or five-level item (Strongly Agree/Agree/Disagree/Strongly Disagree, Definitely Yes/Yes/Maybe/No/Definitely Not, Very Good/Good/Average/Bad/Very Bad, Much Better/Better/The Same—No Change/Worse/Much Worse). Pilot testing of the draft questionnaire was conducted by dialing 20 randomly selected telephone numbers from the national telephone directory, interviewing respondents and completing the questionnaire. Considering the pilot-testing results, the final version of the questionnaire was composed. The list of questions can be found in Table 1.

Data were entered into the database that was developed using the lime survey software [10] of the university-secured server. Anonymity, privacy, and confidentiality were maintained during data collection, entry, analysis, and storage. The study was approved by the Steering Committee of the Postgraduate Program of Applied Public Health and Environmental Hygiene of the Medical Faculty, University of Thessaly (Assembly of April 2020; Project Identification Code 11/2019–2020). Data collection took place from 15 April to 2 May 2020.

Qualitative variables were presented as frequencies with percentages and 95% Confidence Intervals (CI), and quantitative variables were presented with mean and standard deviation. For a univariate analysis, the chi-square test was applied to associate demographic characteristics and other factors with KAP responses, as well as scores of responses to knowledge questions calculating the Relative Risks (RR), with corresponding 95% CI. Multivariable logistic regression models were used to identify independent risk factors for the KAP and scores of responses to knowledge questions to calculate the Odds Ratios (OR) and the corresponding 95% CI. Factors with a *p*-value less than 0.20 in univariate analysis were included in multivariable analysis. A result with a *p*-value < 0.05 was considered to be statistically significant. All statistical analyses were conducted taking into account the clusters of the study through the complex sample module of SPSS 19.0 (IBM SPSS Inc., Armonk, NY, USA). Participants were asked to rate their own income as “Low/Intermediate/High” without considering a specific numerical threshold. KAP were compared among the population of the geographical region of Attica (the major urban area that represents about 35% of the total Greek population) and the other regions of Greece. Three age categories were used in the analysis: 18–44, 45–60, and 61–92. Each correct response to a KAP question was scored with one point. Correct responses in the four-level quantitative items options for answers provided were considered both values Agree/ Strongly agree or Disagree/Strongly disagree depending on the question. Three aggregated scores were calculated: (a) questions 1–12, 14–15 and 22–24 with a maximum score of “17”, (b) questions 1–12 with a maximum score of “12” and (c) questions 14–15 and 22–24 with a maximum score of “5” (questions are listed in Table 1).

A random-digit dialling survey was conducted by 29 interviewers who were trained in communication and data collection methods. To validate the effectiveness of interviewers’ training, results were analyzed per interviewer according to the questionnaire response rate and the missing values per question. No significant associations were found. All candidate respondents were informed regarding the study’s research objectives, the absence of any commercial purposes of the survey, and how their privacy and the confidentiality of data would be ensured. After receiving respondents’ verbal consent to participate in the survey, the interview began by posing the 35 questions. At the end of the survey, respondents were asked about any potential questions they might have in order for interviewers to provide appropriate answers or clarifications.

## 3. Results

Interviewers dialled 27,241 random digits and 12,396 of them corresponded to a telephone number. A total of 5852 did not answer the phone, and 774 were business phone numbers. A total of 1858 (32.2%) individuals responded to the telephone survey, while 3912 refused to participate (most of them due to lack of time and approximately 1.5% of them were COVID-19 deniers).

The demographic characteristics of participants are presented in Table 2. The mean age of participants was 49.2 years (standard deviation: 17.4, minimum: 18, maximum: 92). The majority of respondents (98.3%) held Greek nationality. Fifty-eight of the 1822 respondents (3.2%) had people in their immediate social environment who were infected with severe acute respiratory syndrome coronavirus 2 (SARS-CoV-2), while 11 respondents (0.6%) were confirmed cases.

### 3.1. Knowledge, Attitudes, and Practices

Table 1 presents the responses related to study questions about knowledge, attitudes, and practices.

Regarding attitudes toward a future vaccination, out of the 1811 respondents, 514 (28.4%) would definitely be vaccinated for SARS-CoV-2, 379 (20.9%) would be vaccinated, 216 (11.9%) would not be vaccinated, and 127 (7.0%) definitely would not be vaccinated, while 575 (31.8%) may be vaccinated.

Approximately 39.6% of the 1813 respondents declared that they were not using a face mask to protect themselves from SARS-CoV-2, while 31.9% of respondents sometimes wore a face mask and 28.5% always used a face mask when outside of their home and before entering indoor areas. Moreover, approximately 31% of the 1757 respondents believed that wearing a face mask at the supermarket was not protecting them against SARS-CoV-2.

Concerning attitudes related to travel, of the 1814 respondents, 100 (5.5%) would definitely travel by airplane for their holidays and 196 (10.8%) would travel, while 530 (29.2%) would not travel and 605 (33.4%) definitely would not travel. Furthermore, 383 respondents (21.1%) reported they may travel.

Out of 1785 respondents, 1316 (73.7%) consider information provided about COVID-19 to be sufficient, while 404 out of the 1785 (22.6%) characterized the information they receive to be excessive, and 3.4% rated the information as insufficient.

From the total of 1840 respondents, 1154 (62.7%, 95%CI: 60.5–64.9) received a score of ≥12 (the maximum score possible if all questions (1–12, 14–15, and 22–24) were answered correctly was 17 points), whereas 916 respondents (49.8%, 95%CI: 47.5–52.1) received a score of ≥9 on questions 1–12 with respect to knowledge, and 943 respondents (51.3%, 95%CI: 49.0–53.5) received a score of ≥3 on questions 14–15 and 22–24 regarding attitudes and practices, respectively (Table 1).

*When performing analysis to test association among the question items 1, 2, 3, 4, 5, 6, 12, 14, among question items 12 and 14, among 1 and 14, 21, 23, among 4 and 25 and among 13 and 1, 4 and 6, correct answers about COVID-19 droplet transmission correlated positively with correct answers about transmission after touching contaminated surfaces and then touching the eyes (OR:7.76, 95%CI:3.75–15.04), about correct knowledge of symptoms of COVID-19 infection (OR:4.79, 95%CI:2.29–9.24), about asymptomatic transmission (OR:9.41, 95%CI:4.36–19.01), and about hand washing as a prevention measure for the transmission of COVID-19 (OR:15.53, 95% CI:6.35–35.89). Correct knowledge about COVID-19 transmission when touching contaminated surfaces and then touching the eyes positively associated with correct answers about the main symptoms of COVID-19 infection (OR:12.27, 95%CI:6.10–23.93), about asymptomatic transmission (OR:13.92, 95%CI:6.45–28.77), about hand washing as a prevention measure for the transmission of COVID-19 (OR:13.75, 95%CI:5.16–34.11) and about correct handwashing practice (OR:2.40, 95%CI:1.31–4.70). Moreover, positive association was found among correct knowledge of droplet transmission and good practice about face mask wearing (OR:4.36, 95%CI:2.44–7.50) and physical distancing of one or more meters (OR:1.86, 95%CI:1.04–3.57). Correct knowledge about transmission through touching contaminated surfaces and then touching the eyes was positively associated with good practice of hand antiseptic carrying when outside the home (OR:4.00, 95%CI:2.25–7.35). Respondents who believed that measures restricting the movement of persons are effective in preventing the transmission of COVID-19 had significantly higher odds to respond correctly to questions about COVID-19 transmission through respiratory droplets (OR:7.86, 95%CI:4.35–13.75), after touching contaminated surfaces (OR:11.01, 95%CI:5.97–19.90), and asymptomatic transmission (OR:11.16, 95%CI:5.45–22.19).*

### 3.2. Factors Associated with Misperceptions

Multivariable logistic regression models were used to test for an association between the aggregate number of wrong answers (at least one incorrect answer versus no incorrect answers and three or more wrong answer versus less than three wrong answers) and the participants’ characteristics. Moreover, multivariable logistic regression models were used to test for an association between individual question responses with participants’ characteristics, including age ≥45, male gender, residence outside Attica, occupation in the public sector, level of education up to secondary school or Master’s/PhD degree, middle or high income, and the use of ≥2 information sources. Table 3 presents results of the association of participants’ characteristics with misperceptions. Table 4 presents the participants’ characteristics that correlated with correct answers to the individual questions about knowledge, attitudes, and practices.

## 4. Discussion

Our study demonstrated that despite the fact that the majority of participants had a sound knowledge of COVID-19 transmission modes and prevention measures, good practices related to these topics were not reported by participants at the same level. In particular, about 96% of respondents acknowledged that SARS-CoV-2 is transmitted through respiratory droplets, but only 28.5% responded that they always used a face mask when visiting indoor spaces outside of their home (during the survey period, the use of a face mask was mandatory on mass transport and in taxis, medical facilities, supermarkets, and pharmacies) [6]. Moreover, 98.5% of participants recognized that hand washing could help prevent the transmission of COVID-19, but 58% reported washing their hands for less than 20 seconds and 35% did not carry an antiseptic with them when leaving the house. A great majority of respondents (96.8%) recognized that COVID-19 is transmitted when touching contaminated surfaces and then touching their eyes; however, 43% of respondents did not wash their hands before touching their eyes. Other studies in North America, China (>90%), and Taiwan showed that the majority of respondents were knowledgeable about COVID-19 [11,12,13]. Our findings demonstrate that additional surveys are needed to investigate the reasons why sound knowledge does not always translate into or ensure correct practice. Furthermore, the results of such surveys can be used to adjust the current or design new health information campaigns for COVID-19. Campaigns are important to be based on community engagement; it is important to establish multi-sectoral teams at central, peripheral, and local levels that are able to identify the needs of target groups and address any misinformation and disinformation timely [14]. Messages that include real stories in Greece and trusted messengers for each of the target audiences could also play an important role in the effort to change attitudes.

As shown in Table 3, participants aged 18–44 years, male gender, specific occupations (freelancer, unemployed, housewife, retiree), and those who sought information about COVID-19 from less than two sources received lower aggregated scores on knowledge questions. The ECDC’s recent rapid risk assessment report (24 September 2020) highlighted that in several countries, the increasing reported number of COVID-19 cases correlates with high transmission among persons aged 15–49 years as well as with increased testing rates [1]. These epidemiological data and our study findings related to incorrect knowledge among persons 18–44 years of age demonstrate an urgent need for adjusting information campaigns targeting especially people below 45 years of age, in order to sensitize them to realize the role they can play in the spread of the epidemic and the importance of their contribution to control the spread [1]. Serial cross-sectional KAP studies are needed for the general population, as well as focused surveys for groups where minimal knowledge or incorrect practices have been identified [2]. Future studies could measure changes in the KAP of the population before and after governmental interventions.

Respondents in our survey rated their mental health as worse (41.6%) to a greater extent than they rated their physical health (12.6%), when comparing periods before and during the lockdown measures. Similar findings were also identified in other studies conducted in China and Spain [15,16]. Loneliness experienced throughout lockdown measures and anxiety about financial issues can affect mental health during the restrictive measures [1]. In our study, approximately 68% of respondents expected their financial status to worsen after the pandemic. Protection measures for mental health should also be part of the COVID-19 pandemic prevention strategies.

This was an observational study with voluntary participation in the general population with a relatively low response rate and relatively high mean for age of participants (about 49 years), and therefore, generalization of the results for the Greek population cannot be safely assumed. Selection bias and information bias might have occurred. Additional serial cross-sectional KAP studies with bigger sample are needed in order to be more representative for the Greek population. Moreover, it was not possible to test for ethnic or disadvantaged population groups [2].

## 5. Conclusions

In view of scenarios of sustained COVID-19 community transmission in several European countries in the coming months, understanding the perceptions of people and especially those below 45 years of age, their concerns and beliefs, as well as their knowledge and practices related to COVID-19 is essential to target communication strategies so they can be engaged and actively participate in the battle against the COVID-19 pandemic.

## Figures and Tables

**Table 1:** Knowledge, attitudes, and practices reported by respondents in Greece during the nationwide lockdown in April 2020.

Knowledge/Attitude/Practice Questions	Response	Number/Total (%)	95% CI ^1^
Knowledge			
COVID-19 is transmitted through respiratory droplets *	Agree/Strongly agree	1735/1808 (96.0)	94.9–96.8
2. COVID-19 is transmitted through air *	Disagree/Strongly disagree	742/1713 (43.3)	41.0–45.7
3. COVID-19 is transmitted through the consumption of contaminated food *	Disagree/Strongly disagree	951/1644 (57.8)	55.4–60.2
4. COVID-19 is transmitted when touching contaminated surfaces and then touching the eyes *	Agree/Strongly agree	1760/1819 (96.8)	95.8–97.5
5. The main symptoms of COVID-19 infection are fever, cough, and myalgia*	Agree/Strongly agree	1726/1803 (95.7)	94.7–96.6
6. COVID-19 can be transmitted from individuals who are infected but are asymptomatic *	Agree/Strongly agree	1741/1786 (97.5)	96.6–98.1
7. I have to wash my hands before and after wearing a face mask *	Agree/Strongly agree	1747/1818 (96.1)	95.1–96.9
8. I have to wash my hands before and after wearing gloves *	Agree/Strongly agree	1622/1804 (89.9)	88.4–91.2
9. Smokers infected with COVID-19 have a higher risk of exhibiting severe symptoms than non-smokers *	Agree/ Strongly agree	1534/1725 (88.9)	87.4–90.3
10. High-risk groups include males, the elderly, and people with hypertension or diabetes *	Agree/Strongly agree	244/1849 (13.2)	11.7–14.8
11. Individuals who develop respiratory symptoms should be isolated from the rest of their family members *	Yes	1576/1819 (86.6)	85.0–88.1
12. Hand washing can help prevent the transmission of COVID-19 *	Agree/Strongly agree	1801/1828 (98.5)	97.9–99.0
Attitude/perception			
13. I believe that measures restricting the movement of persons are effective in preventing the transmission of COVID-19	Agree/Strongly agree	1720/1819 (94.6)	93.4–95.5
14. I believe that the use of a face mask when visiting a supermarket protects me from COVID-19 *	Agree/Strongly agree	1213/1757 (69.0)	66.8–71.2
15. I believe that in addition to the use of a face mask, safety goggles are essential for protection from COVID-19 in outdoor areas *	Disagree/Strongly disagree	1230/1692 (72.7)	70.5–74.8
16. I believe that fear about the risks of COVID-19 is unreasonable	Disagree/Strongly disagree	1441/1785 (80.7)	78.8–82.5
17. I believe that the measures implemented are disproportionately strict with regard to the risks from COVID-19	Disagree/Strongly disagree	1422/1818 (78.2)	76.3–80.0
18. I would still travel by airplane if I had previously scheduled a trip for my summer vacation	Definitely yes/Yes/Maybe	679/1814 (37.4)	35.2–39.7
19. I believe that restriction of movement measures should be withdrawn immediately to avoid financial consequences	Disagree/Strongly disagree	924/1699 (54.4)	52.0–56.7
20. Should a vaccine be available for COVID-19, I will receive it	Definitely yes/Yes/Maybe	1468/1811 (81.1)	79.2–82.8
Practice			
21. When talking with people outdoors, what distance do you keep from them?	≥1 m	1579/1736 (91.0)	89.5–92.2
22. Do you wash your hands before touching your eyes? *	Yes	1033/1815 (56.9)	54.6–59.2
23. Do you use a face mask? *	Yes always when entering indoor areas	517/1813 (28.5)	26.5–30.6
24. How long do you wash your hands? *	≥20 s	738/1755 (42.1)	39.8–44.4
25. When leaving the house, do you carry an antiseptic/disinfectant with you?	Yes	1175/1800 (65.3)	63.0–67.4
Questions about self-rating health and financial status			
26. How would you rate your health today?	Very good/good/moderate	1796/1829 (98.2)	94.5–98.7
27. How would you rate your physical health today in comparison to before the coronavirus pandemic?	Much better/better/same	1599/1826 (87.6)	86.0–89.0
28. How would you rate your mental health today in comparison to before the coronavirus pandemic?	Much better/better/same	1064/1823 (58.4)	56.1–60.6
29. How do you expect your financial status to change after the coronavirus pandemic?	Much better/better/same	730/1718 (42.5)	40.2–44.8

^1^ CI: Confidence Interval. Answers bearing an asterisk (*) were scored with 1 point.

**Table 2:** Demographic characteristics of respondents and source of information about coronavirus disease 2019 (COVID-19).

Characteristic	Categories		Frequency (%)
Age	18–45		736 (39.8)
	46–60		602 (32.6)
	61–92		509 (27.6)
	Total		1847 (100.0)
Region	Attica region		659 (35.9)
	Other regions:	Crete, Southern Aegean, Peloponnese, Central Greece, Western Greece, Ionian Islands, Epirus, Thessaly, Western Macedonia, Central Macedonia, Eastern Macedonia, and Thrace	1176 (64.1)
	Total		1835 (100.0)
Gender	Male		756 (41.2)
	Female		1081 (58.8)
	Total		1837 (100.0)
Level of education	Master or Doctor of Philosophy degree		156 (8.6)
	Bachelor degree		864 (47.4)
	Up to secondary school education		802 (44.0)
	Total		1822 (100.0)
Occupation	Civil servant		254 (14.0)
	Private sector employee		424 (23.3)
	Other		1142 (62.7)
	Total		1820 (100.0)
Income	Low		623 (38.2)
	Middle		906 (55.6)
	Higher		100 (6.1)
	Total		1629 (100.0)
Source of information about COVID-19 situation and prevention measures	Television and/or radio		1536 (83.0)
	Family and/or friends		492 (26.6)
	Social media		611 (33.0)
	Websites of a public health institution		366 (19.9)
	Internet		1108 (59.9)
	Physician		342 (18.5)
	Less than two different sources of information		496 (26.3)
	Two different sources of information		633 (34.2)
	More than two different sources of information		729 (39.4)
	Total		1849 (100.0)

**Table 3:** Multivariable analysis of participants’ characteristics and misperceptions about COVID-19.

Participants’ Characteristics and Other Factors		Aggregate Score in the Knowledge Questions * 1, 7, 11, 12, 14, 21			
		At Least One Incorrect Answer Versus No Incorrect Answers		Three or More Incorrect Answer Versus Less than Three Incorrect Answers	
		N/total (%)	Odds Ratio (95%CI)	N/total (%)	Odds Ratio (95%CI)
Age (years)	18–44	381/730 (52.2)	-	26/730 (3.6)	-
	45–60	249/600 (41.5)	0.68 (0.54–0.86)	10/600 (1.7)	0.46 (0.21–0.95)
	61–92	141/509 (27.7)	0.38 (0.29–0.49)	5/509 (1.0)	0.16 (0.05–0.41)
Gender	Male	332/752 (44.1)	-	26/752 (3.5)	-
	Female	434/1076 (40.3)	0.92 (0.75–1.13)	16/1076 (1.5)	0.41 (0.21–0.77)
Region	Attica region	280/659 (42.5)	0.99 (0.80–1.22)	12/659 (1.8)	1.35 (0.69–2.80)
	Other regions	488/1173 (41.6)	-	29/1173 (2.5)	-
Level of Education	Up to secondary school	366/864 (42.4)	-	23/864 (2.7)	-
	Bachelor degree	328/802 (40.9)	0.83 (0.67–1.04)	18/802 (2.2)	1.00 (0.51–1.93)
	Master/PhD degree	71/156 (45.5)	0.88 (0.60–1.28)	1/156 (0.6)	0.29 (0.02–1.47)
Occupation	Public sector	108/254 (42.5)	-	1/254 (0.4)	-
	Private sector	194/424 (45.8)	1.02 (0.74–1.42)	9/424 (2.1)	3.90 (0.71–72.73)
	Other (freelancer, unemployed, housewife, retired, other)	458/1142 (40.1)	1.00 (0.74–1.36)	32/1142 (2.8)	7.45 (1.56–133.69)
Income	Low	268/623 (43.0)	-	19/623 (3.0)	-
	Middle	349/906 (38.5)	0.79 (0.64–0.98)	14/906 (1.5)	0.59 (0.28–1.20)
	High	53/100 (53.0)	1.37 (0.89–2.11)	2/100 (2.0)	0.61 (0.09–2.20)
Number of information sources	<2	179/478 (37.4)	-	18/478 (3.8)	-
	2	265/633 (41.9)	1.01 (0.78–1.33)	7/633 (1.1)	0.20 (0.07–0.49)
	>2	328/729 (45.0)	1.04 (0.80–1.35)	17/729 (2.3)	0.44 (0.21–0.90)
Source of information about COVID-19	Television and radio	607/1536 (39.5)	0.69 (0.52–0.90)	27/1536 (1.8)	0.52 (0.26–1.06)
	Family and friends	220/492 (44.7)	1.06 (0.84–1.33)	11/492 (2.2)	0.83 (0.39–1.64)
	Social media	289/611 (47.3)	1.06 (0.85–1.32)	12/611 (2.0)	0.57 (0.26–1.13)
	Internet	501/1108 (45.2)	1.09 (0.87–1.36)	25/1108 (2.3)	0.70 (0.36–1.38)
	Doctor	134/342 (39.2)	0.86 (0.66–1.11)	7/342 (2.0)	0.95 (0.38–2.06)
	Websites of public health institutions	156/366 (42.6)	0.91 (0.70–1.17)	4/366 (1.1)	0.40 (0.12–1.01)

* The questions are listed in Table 1.

**Table 4:** Participants’ characteristics correlated with correct answers to the individual questions about knowledge, attitudes, and practices.

Factor		Number of Question (Q) *	N/Total (%)	Odds Ratio (95%CI)
Age (years)	18–44	-		
	45–60	Q3	350/544 (64.34)	1.31 (1.02–1.68)
		Q5	565/583 (96.91)	1.85 (1.07–3.34)
		Q8	535/587 (91.14)	1.49 (1.03–2.16)
		Q14	394/576 (68.40)	0.70 (0.55–0.90)
		Q18	212/593 (35.75)	0.55 (0.43–0.69)
		Q19	311/504 (61,71)	1.46 (1.16–1.83)
		Q21	555/581 (95.52)	4.25 (2.75–6.80)
		Q23	157/599 (26.21)	1.53 (1.17–2.00)
	61–92	Q2	181/456 (39.69)	0.74 (0.57–0.95)
		Q3	213/428 (49.77)	0.69 (0.52–0.90)
		Q8	449/490 (91.63)	1.58 (1.07–2.37)
		Q9	436/474 (91.98)	2.11 (1.40–3.24)
		Q13	484/504 (96.03)	1.82 (1.04–3.29)
		Q14	397/479 (82.88)	0.33 (0.24–0.44)
		Q15	246/442 (55.66)	0.30 (0.23–0.39)
		Q17	421/503 (83.70)	1.82 (1.35–2.46)
		Q18	366/725 (50.48)	0.27 (0.20–0.35)
		Q19	187/419 (44,63)	2.12 (1.64–2.74)
		Q20	447/503 (88.87)	2.74 (1.96–3.88)
		Q21	439/458 (95.85)	5.87 (3.52–10.29)
		Q23	210/491 (42.77)	2.93 (2.22–3.88)
		Q26	488/507 (96.25)	0.21 (0.08–0.51)
Gender	Male	-		
	Female	Q2	453/997 (45.44)	1.22 (1.01–1.49)
		Q4	1038/1064 (97.56)	1.81 (1.07–3.09)
		Q7	1032/1063 (97.08)	1.89 (1.17–3.07)
		Q8	970/1057 (91.77)	1.64 (1.20–2.25)
		Q13	1020/1065 (95.77)	1.77 (1.17–2.69)
		Q15	680/977 (69.60)	0.70 (0.55–0.87)
		Q17	856/1062 (80.60)	1.38 (1.10–1.73)
		Q18	324/1063 (30.48)	0.48 (0.40–0.59)
		Q21	932/1008 (92.46)	1.47 (1.04–2.08)
		Q22	648/1059 (61.19)	1.53 (1.26–1.85)
		Q23	340/1056 (32.20)	1.53 (1.23–1.91)
		Q25	787/1045 (75.31)	3.02 (2.43–3.77)
		Q29	453/1008 (44.94)	1.28 (1.03–1.58)
Region	Attica	-		
	Other regions	Q3	577/1057 (54.59)	0.69 (0.55–0.86)
		Q5	1089/1147 (94.94)	0.57 (0.33–0.94)
		Q8	1055/1149 (91.82)	1.79 (1.30–2.45)
		Q10	180/1176 (15.31)	1.80 (1.33–2.47)
		Q15	809/1089 (74.29)	1.29 (1.02–1.62)
		Q25	718/1149 (62.49)	0.74 (0.59–0.94)
		Q27	1050/1162 (90.36)	1.95 (1.47–2.59)
Level of Education	Up to secondary school	-		
	Bachelor	Q10	120/802 (14.96)	1.44 (1.08–1.93)
		Q12	791/797 (99.25)	2.95 (1.24–8.14)
		Q17	631/793 (79.57)	1.33 (1.04–1.69)
		Q18	325/792 (41.04)	1.63 (1.13–2.35)
		Q21	715/767 (93.22)	2.03 (1.41–2.93)
		Q23	227/794 (28.59)	1.29 (1.02–1.63)
		Q25	555/793 (69.99)	1.57 (1.24–1.99)
		Q29	298/752 (39.63)	0.73 (0.58–0.92)
	Master/PhD	Q9	137/143 (95.80)	3.37 (1.56–8.78)
		Q10	26/156 (16.67)	1.74 (1.06–2.76)
		Q12	155/155 (100.00)	Not applicable
		Q18	77/155 (49.68)	1.28 (1.03–1.59)
		Q19	87/136 (63.97)	1.71 (1.17–2.51)
		Q21	141/147 (95.92)	4.43 (2.03–11.67)
		Q25	117/154 (75.97)	2.25 (1.45–3.55)
Occupation	Public sector	-		
	Private sector	Q23	104/420 (24.76)	1.84 (1.23–2.78)
		Q29	155/398 (38.94)	0.63 (0.45–0.89)
	Other	Q23	361/1123 (32.15)	1.96 (1.35–2.89)
		Q29	453/1062 (42.66)	0.68 (0.49–0.93)
Income	Low	-		
	Middle	Q6	875/886 (98.76)	3.57 (1.80–7.58)
		Q11	795/899 (88.43)	1.43 (1.06–1.93)
		Q25	605/892 (67.83)	1.28 (1.01–1.61)
		Q29	378/852 (44.37)	1.45 (1.16–1.83)
	High	Q11	91/99 (91.92)	2.14 (1.07–4.93)
		Q14	56/99 (56.57)	1.72 (1.10–2.68)
		Q29	49/95 (51.58)	2.11 (1.34–3.33)
Number of information sources	<2	-		
	2	Q3	319/564 (56.56)	0.74 (0.55–0.98)
		Q4	613/625 (98.08)	3.26 (1.68–6.74)
		Q6	612/622 (98.39)	2.71 (1.28–6.12)
		Q9	539/599 (89.98)	1.54 (1.04–2.29)
		Q11	553/627 (88.20)	1.65 (1.14–2.39)
		Q12	625/628 (99.52)	5.43 (1.75–23.74)
		Q13	602/627 (96.01)	2.84 (1.68–4.93)
		Q19	334/533 (62.66)	1.39 (1.07–1.81)
		Q20	521/626 (83.23)	1.65 (1.21–2.27)
		Q21	551/598 (92.14)	1.67 (1.05–2.65)
	>2	Q2	278/687 (40.47)	0.73 (0.56–0.94)
		Q3	384/699 (54.94)	0.73 (0.55–0.97)
		Q4	708/727 (97.39)	2.52 (1.39–4.69)
		Q6	702/715 (98.18)	2.40 (1.19–5.03)
		Q9	619/692 (89.45)	1.56 (1.06–2.29)
		Q11	634/720 (88.06)	1.48 (1.04–2.11)
		Q13	691/724 (95.44)	2.41 (1.47–3.97)
		Q20	582/719 (80.95)	1.55 (1.14–2.10)
		Q29	268/687 (39.01)	0.66 (0.51–0.86)

* The questions are listed in Table 1.
